# Understanding the Effects of Repetitive Transcranial Magnetic Stimulation on Neuronal Circuits

**DOI:** 10.3389/fncir.2016.00067

**Published:** 2016-08-23

**Authors:** Natalie A. Matheson, Jon B. H. Shemmell, Dirk De Ridder, John N. J. Reynolds

**Affiliations:** ^1^Department of Anatomy, Brain Research NZ, University of OtagoDunedin, New Zealand; ^2^School of Physical Education, Sport and Exercise Sciences, Brain Research NZ, University of OtagoDunedin, New Zealand; ^3^Department of Surgical Sciences, Dunedin School of Medicine, Brain Research NZ, University of OtagoDunedin, New Zealand

**Keywords:** rTMS, transcranial magnetic stimulation, plasticity, electrophysiology, cortex excitability, neuromodulation

## Summary

Despite the widespread use of repetitive transcranial magnetic stimulation (rTMS) in both research and clinical settings, there is a paucity of evidence regarding the effects of its application on neural activity. Studies investigating the effects of rTMS on human participants (Huang et al., 2005) have shown that patterned trains of rTMS can be used to modulate the sensitivity of motor pathways for a period outlasting the stimulation itself. These changes are often attributed to an rTMS-induced increase in neural “plasticity” or a “change in excitability” of the motor pathway. Evidence that rTMS can modify the strength of motor pathways has led to its introduction into stroke rehabilitation research. It is hypothesized that post-stroke, rTMS can enhance plasticity induction within the brain and, when combined with manual therapy, can facilitate surviving neurons assuming the function of those lost to the stroke (Hsu et al., 2012). In practice however, despite a multitude of studies investigating this approach, there remains no convincing evidence that rTMS is capable of promoting sustained long-term improvement in recovery, above the effects of rehabilitation alone (Hsu et al., 2012; Lefaucheur et al., 2014). We are of the opinion that a lack of advancement within the field is due to an incomplete understanding of the effects of TMS on neural elements. Here we discuss some of the existing evidence and propose experimental approaches that may enhance the human application of rTMS.

## Motor evoked potentials and interpretation of changes in excitability

A vast number of studies attempting to understand the effects of rTMS in humans utilize muscle potentials evoked by single cortical stimuli [motor evoked potentials (MEPs)] as the primary measure of changes in neural activity. Studies using MEPs to measure changes in the responsiveness of cortico-motor pathways however, cannot determine the source of observed changes, nor the manner in which individual neurons contribute to the overall effect. Often a “change in excitability” of motor pathways is described when MEP size is altered following rTMS. This term is somewhat misrepresentative however, as it is likely that changes in MEP amplitude represent a hybrid of changes of the intrinsic excitability of neurons within the activated pathway and alterations in the strength of the connections between these neurons. These processes occur through different mechanisms (Mozzachiodi and Byrne, 2010), with the response of the motor pathway to rTMS protocols critically determined by the extent to which neuronal excitability and synaptic plasticity are induced.

It is important that we clearly understand the neural adaptations induced by different rTMS protocols. Considering limitations with current technology for human investigation, combining electrophysiological recordings with magnetic stimulation in animal experiments will enhance our understanding of how TMS alters the state of stimulated neurons. As with all research using animal models, there are limitations when attempting to translate results into humans. Animal research has predominantly focused on the effects of rTMS on primary motor and visual cortices, because evoked responses from these areas are well characterized and easy to obtain. rTMS however is often applied to frontal cortical regions in humans suffering from psychological conditions such as depression. Differences in the microstructure and surface curvature between primary and non-primary regions may lead to altered responses to rTMS. In addition, such differences may be more prominent between animals and humans, as well as the presence of anesthesia in animals and absence in humans, which in total may influence the applicability of animal model results to human research. This work however is critical in providing a starting point for investigation into the effects of TMS on the brain.

## Electrophysiological evidence of effects of single pulse TMS

Electrophysiological studies in animals facilitate a deeper, more mechanistic investigation into the effects of TMS pulses on circuits within the brain. Recordings of both single and multi-cell activity provide insight into neural activity changes immediately following magnetic stimulation. The parameter space within which TMS, in particular rTMS, operates is vast, with factors such as coil orientation (Brasil-Neto et al., 1992), stimulation intensity (Pascual-Leone et al., 1994), and brain activation state (for review see Silvanto and Pascual-Leone, 2008) all playing a role in influencing the effects of TMS on neural activity. The number of possible permutations of these factors presents a challenge for those attempting to understand the mechanisms of TMS action. The application of animal models provides an opportunity for many of these permutations to be investigated without the need to apply TMS to human participants over prolonged periods of time, reducing confounding effects of attention span and fatigue of the subject.

Single magnetic cortical stimuli induce variable neural responses in extracellular neuronal recordings, which are dependent on stimulation intensity. Mueller et al. (2014) recorded extracellular single unit activity in awake monkeys and observed action potential firing within 1 ms of the onset of a single pulse of TMS. Response to a TMS pulse varied between neurons, but the overall population response showed TMS-induced increases in activity lasting at least 100 ms, with larger increases in spiking rates observed at higher stimulus intensities. Moliadze et al. (2004) also report a transient increase in spontaneous activity following single TMS pulses. Using single pulses applied to the visual cortex of anesthetized cats, Moliadze et al. (2004) showed a stimulus intensity-dependent increase in spontaneous single unit spiking during the 500 ms immediately following the TMS pulse, followed by a sustained depression of activity. Patterns of activation observed between individual neurons were, however, very complex and variable.

Using voltage sensitive dye imaging, also in cat visual cortex, Kozyrev et al. (2014) observed a similar pattern of transient activation (20 ms) followed by suppression of activity lasting 250 ms and then rebound firing. Using short trains of TMS (1–4 s), Allen et al. (2007) and Pasley et al. (2009) showed an increase in single unit spontaneous spike activity immediately following TMS, however in contrast to the previous studies reviewed, this facilitation lasted much longer—approximately a minute. Taken together, these studies report generally similar response patterns to single TMS pulses, however the duration of spike facilitation and suppression periods differs substantially between studies. This is important for the design of rTMS protocols, as the effect of consecutive pulses within a TMS train will depend on the state of the neural circuit at the time of pulse delivery. It therefore becomes critical to know the timing and direction of changes in activity in order to maximize the effects of the rTMS train.

The influence of brain state on the response to single-pulse TMS was clearly shown by Moliadze et al. (2004) who demonstrated that single unit activity in the visual cortex evoked by single-pulse TMS is modulated by the timing of a visual stimulus. A TMS pulse applied several 100 ms prior to the onset of visually evoked activity suppressed spiking rates. However, a TMS pulse delivered immediately prior to the onset of visual-evoked activity facilitated activity, suggesting sub-threshold activity was lifted above threshold. In contrast, Pasley et al. (2009) observed a large TMS-induced reduction of subsequent evoked activity in trials following short train TMS. These results suggest that the effects of TMS are highly dependent upon the instantaneous state of the stimulated neuron, with TMS differentially affecting spontaneous activity and activity induced by other converging afferent signals.

The intensity of TMS may be a critical determinant of which cortical neurons will fire in response to each stimulus, due to differences in membrane properties. Neurons with a low firing threshold, such as certain subtypes of GABAergic interneurons (Kawaguchi and Kubota, 1997), may be preferentially activated at low TMS intensities while higher threshold neurons remain silent (Moliadze et al., 2005). Earlier activation of inhibitory neurons likely renders cortical output neurons less responsive to subsequent stimuli. In contrast, higher intensity conditioning stimuli may also recruit higher threshold excitatory neurons, leading to a facilitated response to subsequent TMS pulses. Many questions still remain, for example, do specific intensities and orientations of the stimulation field target specific neuronal types or layers of the cortex? The answer to important questions such as this can be answered using precise electrophysiological techniques.

## Electrophysiological evidence of effects of repetitive TMS

Despite an incomplete understanding of the mechanisms of action of single TMS pulses, the promise of non-invasive neuromodulation for treating cognitive and motor disorders has spawned a large number of investigations of the effects of rTMS. The application of repeated TMS pulses adds additional layers of complexity in determining its effects on the brain. Adding multiple pulses together to form a stimulus train introduces additional parameters, such as stimulus-train length, frequency, number of pulses delivered, and their temporal pattern. All of these parameters may differentially affect the responses of the intended target neurons to rTMS.

A systematic study investigated the effects of nine different protocols on visually evoked potential (VEP) amplitude (Aydin-Abidin et al., 2006). Protocols delivered at 1, 3, and 10 Hz, were delivered for 1, 5, or 20 min and each contained a different number of pulses. VEP amplitudes were differentially altered depending on the protocol applied. Overall, the high frequency trains (10 Hz) were more effective at exerting effects within short periods of time whilst low frequency stimulation took longer to exert an effect. Correspondingly, 10 Hz stimulation delivered by Kozyrev et al. (2014) led to a build-up of an excitatory state with each pulse in the 10 Hz train leading to a “stepwise increase in cortical activity” (Kozyrev et al., 2014). rTMS in this experiment was delivered as 5 blocks of 5 pulses delivered at 10 Hz, with each block separated by a 7-s inter-stimulus interval. What is not clear was which of the components of the stimulation primarily contributed to the overall excitation, i.e., the 10 Hz frequency or the patterned delivery of five blocks of stimulation. Benali et al. (2011) have provided evidence that the pattern of stimulation may be the more critical parameter. They reported that high frequency pulses (50 Hz) delivered in a continuous manner have no effect on multi-unit firing rate, while delivering 50 Hz stimulation in bursts, increases firing rates for several hours. Such observations may contribute to the effects of specific burst patterns such as theta burst (Huang et al., 2005) and quadripulse stimulation (Hamada et al., 2007).

The intensity of rTMS is critical to the resulting effects on neural tissue, as it is for single pulses. Ogiue-Ikeda et al. (2003) delivered 25 Hz rTMS over 7 days to non-anesthetized animals at different intensities and tested hippocampal long-term potentiation (LTP) in *ex vivo* slices. They found that increasing rTMS intensity resulted in a falloff in LTP then a suppression at high intensities which is somewhat surprising and could occur as a result of rTMS-induced damage to the brain. This highlights the need for additional investigation of the effects of rTMS intensity in animal models, and also demonstrates that rTMS applied to the cortex can influence the activity of subcortical structures such as the hippocampus. The latter was also displayed by Ahmed and Wieraszko (2006) who described changes in hippocampal LTP and subsequent changes in memory retention, in mice administered cortical rTMS. In human subjects, direct hippocampal activation would not be expected, due to the inability of the rTMS-induced magnetic field to penetrate the large distance between cortex and hippocampus. It is unclear however, how the gross effects of rTMS may lead to changes in distant brain regions. Evidence in animals that rTMS alters activity in non-targeted areas suggests this is a factor that requires careful consideration and additional investigation.

## Challenges for the future

Electrophysiological studies such as those reviewed, provide much needed information on the effects of TMS pulses on neural responses. This information is an important step toward a clearer understanding of the effects of rTMS and the design of more efficacious protocols. What these studies do not provide however is an insight into the changes in intrinsic excitability and synaptic plasticity that may be occurring in the intact brain following rTMS. In order to obtain this kind of information, a different approach is required.

Intracellular recording techniques provide information on both changes to neuron membrane properties that indicate alterations in intrinsic excitability and information on the strength of synapses in the recorded pathway. Unfortunately, the complexity of recording intracellular responses during TMS has made this a significant technical challenge (Matheson et al., 2015). Dissecting the type of plasticity that is occurring in response to different parameters is critical when considering the design of an rTMS paradigm. Altering the intrinsic excitability of neurons will likely result in changes in homeostatic processes within the brain. For example if neuronal excitability is increased, then homeostatic regulation may occur to ensure the circuit is less responsive to subsequent activation, in order to avoid cascades of hyper-excitation. In the clinic, this may mean that motor rehabilitation tasks performed following an excitatory rTMS protocol may show little benefit to functional recovery. To design maximally effective protocols, for treatment of human neurological and psychiatric disorders such as stroke and depression, respectively, our aim should be to understand the cellular effects of rTMS-induced plasticity and the changes in activation dynamics it induces. The large parameter space can be bridged by computational modeling approaches (e.g., Wilson et al., 2014, 2016). There is an urgent need for researchers in pre-clinical and clinical settings to combine forces and share openly and completely the results of experimental techniques (Héroux et al., 2015), with the focus on developing clinically effective rTMS protocols.

## Author contributions

NM drafted the manuscript. All authors critically reviewed and revised the manuscript and approved the final submitted version.

## Funding

NM was supported by a University of Otago PhD scholarship. JR received support from a Rutherford Discovery Fellowship from the Royal Society of New Zealand.

### Conflict of interest statement

The authors declare that the research was conducted in the absence of any commercial or financial relationships that could be construed as a potential conflict of interest.
